# A framework for the management of donated medical devices based on perspectives of frontline public health care staff in Ghana

**DOI:** 10.1177/2399202620941367

**Published:** 2020-09-18

**Authors:** Dinsie B Williams, Jillian C Kohler, Andrew Howard, Zubin Austin, Yu-Ling Cheng

**Affiliations:** 1 Leslie Dan Faculty of Pharmacy, University of Toronto, Toronto, ON, Canada; 2 Munk School of Global Affairs, University of Toronto, Toronto, ON, Canada; 3 WHO Collaborating Center for Governance, Accountability and Transparency in the Pharmaceutical Sector, University of Toronto, Toronto, ON, Canada; 4 Department of Surgery, Faculty of Medicine, University of Toronto, Toronto, ON, Canada; 5 Division of Orthopaedic Surgery, The Hospital for Sick Children, Toronto, ON, Canada; 6 Centre for Global Engineering, University of Toronto, Toronto, ON, Canada; 7 Department of Chemical Engineering and Applied Chemistry, University of Toronto, Toronto, ON, Canada

**Keywords:** Access, health care, donations, good governance, medical devices, philanthropy, policy, Ghana, medical devices management

## Abstract

**Background:**

Transnational funders provide up to 80% of funds for medical devices in resource-limited settings, yet sustained access to medical devices remains unachievable. The primary goal of this study was to identify what factors hinder access to medical devices through the perspectives of frontline public hospital staff in Ghana involved in the implementation of transnational funding initiatives.

**Methods:**

A case study was developed that involved an analysis of semi-structured interviews of 57 frontline technical, clinical and administrative public health care staff at 23 sites in Ghana between March and April 2017; a review of the national guidelines for donations; and images of abandoned medical devices.

**Results:**

Six key themes emerged, demonstrating how policy, collaboration, quality, lifetime operating costs, attitudes of health care workers and representational leadership influence access to medical devices. An in-depth assessment of these themes has led to the development of an enterprise-wide comprehensive acquisition and management framework for medical devices in the context of transnational funding initiatives.

**Conclusion:**

The findings in this study underscore the importance of incorporating frontline health care staff in developing solutions that are targeted at improving delivery of care. Sustained access to medical devices may be achieved in Ghana through the adoption of a rigorous and comprehensive approach to acquisition, management and technical leadership. Funders and public health policy makers may use the study’s findings to inform policy reform and to ensure that the efforts of transnational funders truly help to facilitate sustainable access to medical devices in Ghana.

## Background

According to the World Health Organization (WHO), medical devices are essential to all health care systems.^1^ A medical device is any instrument, apparatus, implement, machine, appliance, implant, in vitro reagent or calibrator, software, material or other similar or related article that does not achieve its primary intended action in or on the human body solely by pharmacological, immunological or metabolic means. Yet, public health care providers in resource-limited settings commonly lack sustainable access to medical devices that they need to effectively and efficiently diagnose, treat and monitor their patients.^2^ For example, public health care clinicians in Ghana have less than 10% of the medical devices that they need to deliver essential services. Recommendations for capacity are based on the list of items considered essential by the Ghana Health Service (GHS) Emergency Supply Checklist or the WHO’s Guidelines for Essential Trauma Care or Integrated Management for Emergency and Essential Surgical Care (IMEESC) tool kit.^3–6^ The Government of Ghana reports having four computed tomography (CT) scanners and two magnetic resonance imaging (MRI) scanners that serve a population of 26 million people,^3^ while across 25 countries in the Organisation for Economic Co-operation and Development, there is an average of 23.5 (range: 8.4–59.6) CT scanners and 15.1 (range: 2.4–39) MRI scanners per million people.^7^ In an assessment of availability of electronic medical equipment in 40 hospitals across Ghana in 2014 and 2015, 87.5% had no X-ray scanners and 80% had no small-volume diagnostic laboratory sampling devices for use in paediatric trauma care.^4^ Although the number of devices per capita may have increased recently, access to functioning devices is still limited. Without adequate medical devices, health care providers are unable to diagnose, monitor or treat their patients effectively and efficiently.^2^ The indirect impact of the shortage in diagnostic laboratory devices such as microscopes in Ghana was demonstrated by the findings from a study which reported that out of 689 children who were presumptively diagnosed with malaria, the introduction of microscopy revealed that only 53.6% had the disease.^8^ In addition, microscopy detected malaria in 14.2% of children who were previously determined to be free of malaria.^8^

The Government of Ghana, like other governments in sub-Saharan Africa, relies on non-public funding sources, including patients and transnational funders, to address the shortage of medical devices. Transnational funders are groups that facilitate the transfer of funds and goods across national borders to meet philanthropic, development or financial goals. Transnational funders include individual philanthropists and private corporations, faith-based entities such as religious organizations, government agencies and development partners, and non-governmental organizations. They often financially support public health care systems that are unable to provide adequate health care to their citizens. In fact, transnational funders contribute as much as 80% of the money spent on equipping health care systems in resource-limited settings.^9^ Governments that rely heavily on transnational funding, specifically official development aid, have challenges managing the funding processes.^10^ The governments cannot predict incoming funding appropriately and they often lack governance mechanisms, specifically those related to maintaining accountability.^10^ Despite the availability of transnational funding, sustained access to medical devices remains challenging in resource-limited settings and health care systems experience difficulties in making suitable investments.

As much as 72% of the available medical devices are unused and abandoned. For every US$1 donated toward medical devices, 25 cents come from internally generated funds. If 72% of devices are abandoned, that means 90 cents out of US$1.25 go to waste. If all devices that are abandoned come from donated funds, this means that 90% of donations are wasted.^9,11,12^ To minimize this type of waste, leading policy researchers have called on governments like that of Ghana to develop mechanisms to facilitate governance (specifically transparency and accountability) of transnational funding practices.^13–20^ To develop such governance mechanisms, policy makers need to understand the experiences and perspectives of stakeholders who are involved in transnational funding processes and in the operation and maintenance of the devices.^14^

To this end, this article identifies the factors that hinder access to medical devices by eliciting the perspectives of frontline public health care staff in Ghana who are involved in the implementation of transnational funding initiatives. Funders and public health policy makers may use the study’s findings to inform policy reform and to ensure that transnational funders truly facilitate sustainable access to medical devices in Ghana.

## Methods

### Case study selection

We conducted a case study guided by the research objective to understand how frontline public hospital staff in Ghana perceived transnational donations of medical devices.^21^ Ghana is a country of 26 million citizens with an average life expectancy at birth of 61 years (compared to the global average of 71 years)^22^ and a human development index of 0.57 (compared to the global average of 0.70 ± 0.16).^23^ Four hospitals provide tertiary care, while there are primary care hospitals and health centres in almost all of its 110 districts.^4^ About 49% of Ghana’s health care budget comes from non-public sources, yet common diseases such as malaria continue to be misdiagnosed because of a lack of access to functioning medical devices.^24^

### Data collection

Semi-structured interviews with 57 frontline hospital staff were held at 23 sites (22 public hospitals and 1 regional medical store) between March and April 2017. Interviewees (or key informants) were recruited using a snowball sampling technique. Three individuals identified potential interviewees from within their networks. Those key informants in turn were asked to identify others in their networks. The interviews took place in the Greater Accra, Brong-Ahafo, Ashanti, Eastern, Volta and Central regions. Of the 22 hospitals, 17 were operated by faith-based organizations, while 5 were operated by the government. The interviews were conducted at the locations where the informants normally worked. Key informants’ occupations were distributed as shown in Table 1.

**Table 1.** List of key informants.

Group	Occupation	*n*
Technical	Biomedical scientists, laboratory managers, radiographers, clinical engineering managers, technicians or facilities managers	23
Administrative	Heads of administrative services, medical directors (including practicing physicians) and accountants	17
Support	Procurement officers and store managers	9
Clinical	Anaesthetists and nurses	8

All key informants had a working knowledge of English and voluntarily gave informed written consent to provide information. None of the informants was compensated in any way. All interviews were conducted in English under the assurance that identities of the informants and their locations would not be disclosed. As best as possible, and without changing the context of the responses, evidence of nationalities of funders, brand names, distributors and manufacturers of devices was redacted from the quotes of informants.

In parallel with the interviews, information was collected through direct observation. For this study, direct observation took the form of site visits where images (photographs) of devices in their specific setting were captured using a digital camera. In addition, a review of the national guidelines relevant to medical donations was conducted.

### Data analysis

Thematic analysis of transcriptions of the interviews helped us to identify concepts that demonstrated key informants’ perceptions of transnational funding policies and practices. Transcripts of the screened interviews were deductively coded independently by two reviewers with five *a priori* codes (and 11 sub-codes). The primary codes were value,^25^ standards,^9^ communication,^9^ participation and consensus-orientation^26^ and responsiveness.^26^ These codes were extracted from the conceptual framework of the study which, in turn, was derived from the WHO’s *Guidelines for Healthcare Equipment* Donations,^9^ the Health Policy Analysis framework^25^ and the Governance in Health Systems of Developing Countries framework.^26^ Three codes, control, trust and resource management, emerged from the data and were added inductively. Emergent codes are particularly interesting because the incidental nature of their discovery suggests novel information that is context-sensitive.^27^ These codes were not identified deductively from existing theories. The emergent codes helped us to modify our conceptual understanding of the subject matter inductively.^28^ The finalized structure of codes or codebook is displayed in Table 2. During tours of the sites where interviews were conducted, we captured images of devices and documented the conditions under which they were found. Devices were documented under six categories, based on their condition: (1) terminally inoperable due to obsolescence of parts or components, (2) repairable but in need of components, (3) incompatible with the infrastructure or in need of technical modification, (4) operating under inappropriate conditions, (5) redundant or otherwise not needed and (6) fully functional and in use. A critical review of the Government of Ghana’s *Guidelines for Donations and Voluntary Medical Outreach Programmes in the Health Sector of Ghana* was also conducted. The review involved an assessment of the document’s use of plain language and formatting and its comprehensiveness.^29^ Through a triangulation process, the themes from the interviews were cross-referenced with information from the document review and images of abandoned devices.^30,31^

**Table 2.** Codebook

Primary code	Subcode	Description
Value	Appropriateness: demonstrates that actions are relevant to key informants’ needs or expectations	Suggestion that expectations of key informants are taken into consideration (e.g. completion of needs assessment). Evidence that donations are relevant to the patient population.
	Quality/condition	Indication of the condition of donated items
	Functionality	Indication of whether donated items are in working order or not
Standards	Awareness of policies, government and administrative arrangements	Demonstration of awareness of established rules and adherence to/disregard for the rules
	Availability of documents	Suggestion that key informant has access to a document that describes relevant policies or government and administrative arrangements
	Description of practice	Demonstration of knowledge of events that take place in practice (irrespective of the availability of policy documents)
Communication	Information dissemination	One-way distribution of information
	Information exchange	Reciprocal sharing of information
Participation and consensus-orientation	Collaboration	Demonstrations of cooperation
	Consultation	Inclusion in decision-making or implementation
Responsiveness	Thoughtfulness	Display of empathy or lack thereof. Going beyond the call of duty to ensure that key informants’ needs are met. Exceeding the expectations of key informants.
Emergent primary code	Subcode	Description
Control	Lack of awareness of policies and other information	Acknowledgement of power
Trust	False representation Misplaced priorities Political capital Political relationships	Expressions of trust, or lack thereof, in counterparts’ motivations
Resource management	Planning for: Operations (human resource, consumables, electricity, water), wear and tear (preventive and reparative), and obsolescence (disposal and replacement)	Limit mismatch between devices and available infrastructure

The University of Toronto (Protocol No. 32234) and the Government of Ghana’s Health Service Ethics Review Committee (Approval No. GHC-ERC 09/03/16) approved the study.

## Results

Following analysis of the data from the interviews, six main themes emerged. Representative quotes from key informants are provided here (with redactions as indicated). Three themes emerged deductively from the data and as such were not derived directly from the codebook. Some of the themes were reinforced by observations made at various facilities and through a review of guideline documents.

### Theme 1. Policy – transnational funding guidelines are inadequate and obscure

The critical review of guidelines and data from the interviews provided a consistent picture of the gaps in relevant policies. From the accounts of public health care staff, it was unclear how the Government of Ghana’s *Guidelines for Donations and Voluntary Medical Outreach Programmes in the Health Sector of Ghana* were helping to improve the quality of health-related donations and voluntary medical outreach programmes for the benefit of health care providers and patients. Though clearly written and easy to understand, the guidelines had gaps regarding information that was needed to safeguard the quality of medical devices. The document contained requirements for medicines such as viable shelf-life and specifications for storage and refrigeration, but it was missing equally important requirements for devices, including electric power specifications, operator and patient safety standards, installation and maintenance requirements and disclosures of estimates of lifetime operating costs. Without these specifications, funders and frontline staff had insufficient guidance to select appropriate medical devices and to ensure that they remained useful.

Furthermore, the document did not have instructions on how to promote its use; therefore, many frontline staff were unaware of it. Of the 57 people interviewed, only 8 heads of administrative services or managers were aware of the national guidelines, and only 1 had a copy of the document readily available for use as a reference. When asked about the availability of policies on externally funded devices, the head of administrative services at site 19 presented a copy of the national guidelines and stated that ‘*There is no policy at the hospital. Whatever comes in we accept. But here is the one from the government*’. A head of administrative services at site 16 who was aware of the national guidelines stated, ‘*Yes . . . there is one at the government level [of] which [I] am aware, [it] is with the ministry of health*’. The remaining frontline staff were unaware of the guideline document. Their responses to questions about the guidelines include ‘*There is none*’ (laboratory manager at site 5) and ‘*. . . may be a policy. I am sure there is a policy but as to how it is, I don’t know*’ (laboratory technologist at site 22).

### Theme 2. Collaboration – purposive funding yields benefits

The second theme underscored the benefits of collaboration and the value of donations that target specific needs. Recipients found donations to be more valuable when funders approached the donation process with purpose. Signs of purposive funding include proactive needs assessments and formation of multi-year partnerships. A manager at site 20 stated that ‘[devices that] *come from donors who ask about our needs are useful*’. The head of administrative services at site 15 suggested that ‘*if it is a regular partner, somebody who is providing what you need to use jointly with him, surely . . . there is always a higher chance that it will meet the need*’.

Informants in a variety of occupations ranging from laboratory managers to heads of administrative services highlighted the benefits of functional devices. The laboratory manager at site 1, the facilities manager at site 5, the head of administrative services at site 14, the store manager at site 16 and the senior radiographer at site 21 appreciated receiving functioning, externally funded devices. With chemical analyzers, X-ray scanners and ultrasound scanners, for example, that were in working order, hospital heads of administrative services could replace obsolete models, own devices that they would otherwise not afford, purchase other items or lower the cost of health care to patients. Functioning devices allowed hospital staff to be more efficient and to expand their service offerings. The head of administrative services (at site 21) highlighted secondary benefits beyond the obvious health care benefits: individuals who received treatment could earn money and reduce social conflicts, which in turn ensure ‘*national cohesion and stability*’. Recipients of functioning devices were eager to receive more externally funded devices, while those with incomplete, faulty or otherwise inoperable devices were not.

### Theme 3. Quality – ‘white elephant’ donations

Some funders left behind devices after completing medical outreach missions, without verifying that the hospital had the financial or human resources to manage the devices. The funders did not account for how the devices would be maintained without mission staff. Hospital staff were unable to deliver care with these devices, along with others that did not target a specific clinical need. These so-called ‘white elephants’ littered all hospitals that were visited during this study. One laboratory technician stated that

This chemistry analyzer for example has more capacity than the one we are using but it has been a white elephant because we do not know how to operate it. Some of the boxes have incubators and others are in the store. These have been here for two or three years. And others in the store have been here [for more than five years].

**Figure 1.**
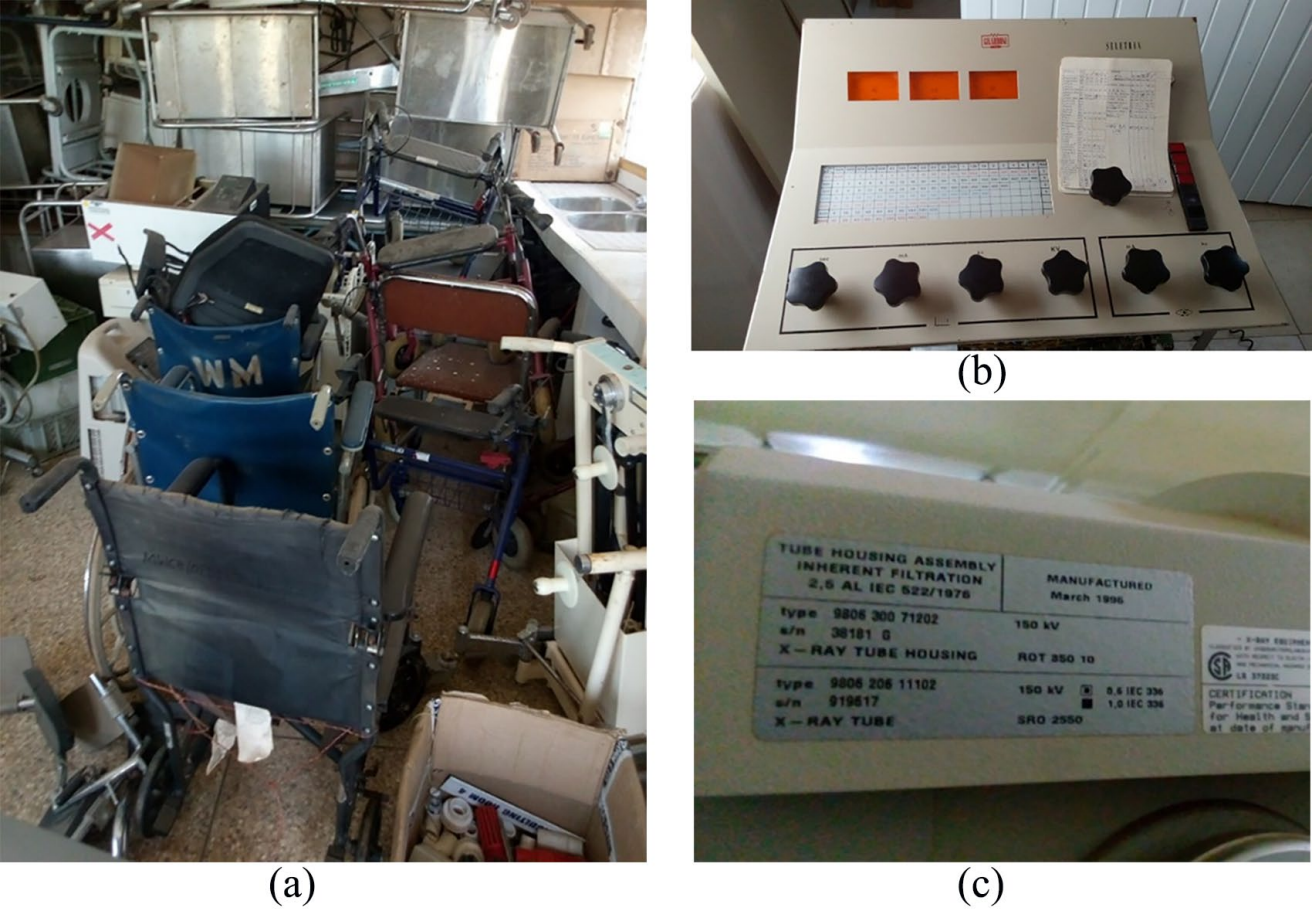
Obsolete or otherwise terminally inoperable devices: (a) multiple obsolete or defective devices, (b) multiple obsolete or defective devices, (c) X-ray console without identifying labels, (d) X-ray tube manufactured in 1996 and (e) manufacturer label of 115 V ultrasound scanner.

**Figure 2.**
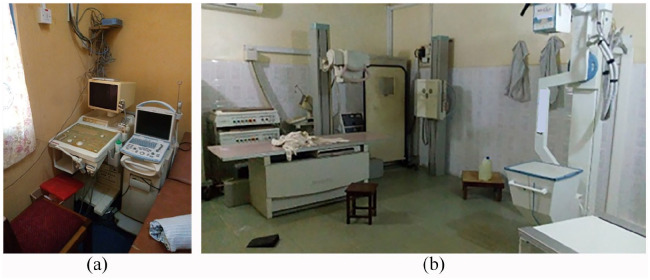
Inappropriate use of devices: (a) redundant ultrasound scanner operating in a location with inadequate airflow and (b) two X-ray scanners installed in the same room.

The pictures that we acquired during direct observation showed abandoned devices and substantiated the accounts of key informants. As shown in Figure 1(a)–(c), there were obsolete, redundant and electrically incompatible devices (e.g. wheelchairs, and oxygen concentrators) that were abandoned at hospital sites, and as shown in Figure 2(a) and (b), there were redundant and otherwise unnecessary, unused devices.

### Theme 4. Lifetime operating cost – the Trojan horse side effects

Some donated medical devices may be abandoned when their lifetime operating costs exceed the host hospitals’ budgets. Replacing parts, purchasing consumables, maintaining the quality of the devices and training operators – these are all operating costs that can grow significantly over the lifetime of a device. Recipients typically do not anticipate these costs which essentially are Trojan horse–like side effects of donations – they are detrimental to the recipients. Replacement parts and consumables interestingly represent revenue opportunities for manufacturers and distributors while draining the meagre resources of recipients. Closed-system analyzers, for example, use specific reagents sold by select distributors (and by proxy, manufacturers). This means that the host hospital must enter into exclusive purchasing agreements with the distributors. Occasionally, a transnational funder (or a group of funders) covers the costs of laboratory reagents for the duration of a health-related initiative. At the end of the initiative, hospital heads of administrative services often find themselves unable to take on the responsibility of purchasing their own consumables at non-competitive prices. Here are two of the key informants’ accounts regarding the impact of externally funded closed-system analyzers:

Without access to funds for replacement parts, consumables and adequately trained technical staff, the primary choice left to heads of administrative services in hospitals is to abandon the devices. We have the [brand name], a full blood hematology analyzer, which was donated through a partnership between the government of Ghana and [name of company]. So they were giving us the reagents for free until the contract was somehow terminated and we could no longer get the reagent. So every facility had to arrange to get the reagent directly from the supplier but it was very expensive. Some facilities were not able to finance the purchase of the reagent. So it became like a wasted whatever [i.e., the equipment went to waste]. (Laboratory manager at site 11)

The reagent [for the biochemical analyzer] can only be sourced through donor but donor project has ended. We did not know the program had ended until we stopped receiving the reagent . . . They should have long-term procurement agreements with the government. Donors have incomplete missions. If you cannot sustain something don’t even start it. (Laboratory manager at site 5)

Along with consumables, preventive and reparative maintenance services are also costly side effects of acquiring medical devices. When external funders do not cover service agreements, recipient hospitals must hire service personnel to maintain the quality of the devices at their own expense. As with replacement parts, it is costly to find and employ technical personnel with biomedical training in Ghana. Despite often amounting to only a fraction of the capital cost of the devices, preventive and reparative maintenance costs are prohibitive to several hospitals. Heads of administrative services usually rely on unpredictable coverage by government-employed service personnel and electricians in place of biomedical technicians or engineers who are trained to work on specific brands of devices. The human resources are inadequate to care for the devices that are being funded.

### Theme 5. Attitude – ‘the beggar has no choice’

A fifth theme emerged deductively that suggested a link between the attitudes of key informants towards funders and the abundance of ‘white elephant’ donations in Ghana. Given that they were reliant on funders to provide them with costly medical devices, some recipients believe that they have no choice other than to accept all externally funded medical devices. They have internalized the position or attitude of beggars. They made statements such as ‘*we are on the begging side, so we cannot focus on the negative*’ (head of administrative services at site 22), ‘*the beggar has no choice*’ (regional store manager at site 9), and ‘*generally half a loaf is better than none*’ (head of administrative services at site 20). These quotes suggest that recipients in multiple locations show deference to funders even when the devices are of poor quality.

### Theme 6. Representation – technical staff leadership gaps on management teams

The final theme suggests that the concerns and needs of technicians, technologists and engineers are not represented by qualified technical personnel on hospital leadership teams. The laboratory manager at site 12 stated that ‘*most of the time there is no technical person on the management board or procurement team*’. Instead, the hospital head of administrative services represents the technical and facilities management staff. While the head of administrative services may have business acumen, their technical acuity and ability to make decisions regarding medical devices is often ‘*limited*’, according to the head of administrative services at site 15. Technical staff are led by individuals who are not professionally trained in technical fields. Other professionals are led by trained personnel, for example, a nurse manager or head nurse represents nurses, a chief medical officer represents physicians and an accountant represents the procurement and financial teams. In attempting to explain the gap in staffing and leadership, the clinical engineering manager at site 2 pointed out that hospitals were established when populations had to rely wholly on the skills of nurses and physicians. Medical devices played a minor role in the delivery of health care. As a result, hospital management teams did not treat technical roles as ‘*critical*’. Now that medical devices are taking on a more prominent role in health care delivery, it is important for hospital management teams to make room for technical leaders. Gaps in technical leadership at the hospital level emerged as a deductive theme during the interview process.

## Discussion

Some of the themes which emerged from our study (relating to collaboration, quality and hidden operating costs) correspond to themes that have been illuminated in other published studies,^11,32–35^ while the others (i.e. policy, attitude and technical leadership) provide new insights into transnational funding of medical devices. In their study of 28 health facilities in Ghana, for example, Bradley et al.^32^ found that some donated devices are of poor quality (i.e. were faulty and not durable), have wrong voltage specifications or are otherwise inappropriate for the setting in the host hospital. Increasingly, funders are supplying new, unusable devices along with non-functioning devices. These unusable devices do not meet an immediate need or often require the services of professionals who are not available in the host country. The devices needlessly occupy clinical space at the various recipient hospitals much in the same way as devices of poor quality. These so-called ‘white elephant’ devices remain at hospital sites indefinitely without any practical mechanisms for their disposal.^35^ The devices create a burden for recipients, taking up space in storage rooms and clinical areas and increasing the risk of harms in hospitals across the country^11,33,34^ Under circumstances where informants found their medical devices were functional and safe to use, they also indicated that the devices required a financial commitment that often exceeded the budgets of the hospitals. Ownership of medical devices is onerous to hospitals that are unprepared to cover lifetime ancillary or operating costs associated with replacing parts, purchasing consumables, conducting preventive and reparative maintenance, and safely disposing of devices when they are no longer useful.^11^

We identified three new and critical findings relevant to policy, attitude and technical staff leadership representation. First, we found that appropriate policies were lacking or poorly implemented. To improve the quality of transnational donations, the WHO developed its *Guidelines for Healthcare Equipment Donations* that impressed upon funders and recipients the importance of collaboration and communication during donation initiatives^9,11,36^ All potential donations had to be assessed for appropriateness, quality and safety, cost-effectiveness, ease of use, maintenance and conformity with the recipient’s policies, plans and guidelines.^36^ The WHO relies on state governments to establish relevant national policies that support the implementation of its guidelines.^9^ Yet, as of May 2014, only just over half of the countries in the African region reported having such national policies on medical devices donations.^3^ Furthermore, of those countries like Ghana that report that they have national guidelines, it is apparent that some of these guidelines may not be adequate. From all accounts, it is unclear how the government’s *Guidelines for Donations and Voluntary Medical Outreach Programmes in the Health Sector* are helping to advance its objective to improve the quality of health-related donations and maximize benefits in the health sector. The guidelines are outdated and missing important device-relevant specifications. Since the interviews were conducted, a newer *Guideline for Donation of Medical Devices* has been published by the Government of Ghana’s Food and Drugs Authority.^37^ This document includes a section on quality assurance and safety and requirements for low energy consumption, compatibility with available devices, ease of use, availability of consumables, installation and maintenance. Most importantly, the document is linked to legislation.

Another novel theme we found was related to the observation that some recipients showed deference in their attitude to funders because they were reliant on the latter to provide costly medical devices. This theme emerged as important because the ‘beggar’s’ attitude of recipients fostered their acceptance of ‘white elephant’ donations. Finally, a third novel theme highlighted the lack of technical personnel on the management teams of public hospitals. Without the right personnel providing technical leadership, there is no oversight of the acquisition, use and disposal of medical devices. This leaves technicians feeling unempowered and unable to extend the lifetime of the devices.

Various authors have offered suggestions on how to improve transnational funding, but none have provided a comprehensive solution that can be readily implemented. New knowledge from our study may be used to augment the body of suggestions that have been offered by other authors. Dzwonczyk et al.^11^ recommended that governments of recipient countries establish clear donation policies and channel all donations through a central office. They highlight the importance of assessing needs, usability and sustainability of donations, establishing that potential donations are suitable for the recipient’s operating environment and confirming that the donations are functional and meet safety standards established by relevant manufacturers.^11^ They note that obsolescence should be managed and that both funders and recipients are responsible for ensuring that the necessary maintenance structure is available.^11^ Other authors have championed the establishment of reliable infrastructure and a resource management team to support externally funded devices.^35,38^ While it is intuitive that improvement in infrastructure can enable hospitals to benefit from medical devices,^35^ post hoc development of infrastructure (material and human resources) around medical devices is inefficient and unsustainable. To minimize avoidable breakdowns, it is necessary to enhance the users’ knowledge of the devices’ underlying technologies and maintenance requirements.^35^ Training has also been highlighted as an important element of the funding process.^32,38^ Unfortunately, the requirement for human resources capacity is an essential component of clinical engineering that is widely misunderstood in the peer-reviewed and popular literature. Training technicians on the basics of operating and troubleshooting devices at installation may be inadequate to foster long-term functionality. An electrical technician without extensive experience or understanding of biomedical technology cannot sustainably serve as proxy to biomedical technicians or service engineers. For complex medical devices, a technician would need access to licensed, proprietary technical information and remote technical support from multiple manufacturers. Considering the accounts of key informants and building upon the published literature, we offer a proposal for enhancing the impact of transnational funding on facilitating sustainable access to medical devices in resource-limited settings.

### A framework for enhancing transnational funding initiatives involving medical devices

**Figure 3.**
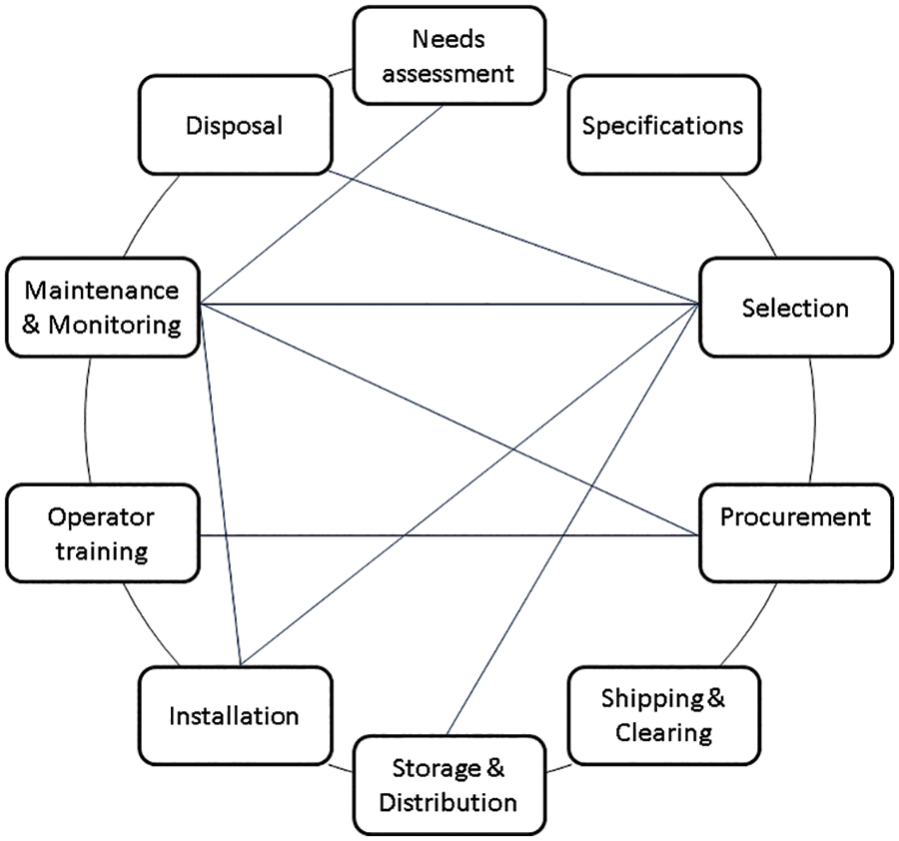
An enterprise-wide comprehensive acquisition and management (CAM) framework for medical devices. Straight lines suggest interdependence between two elements of the framework. *Source*: Ankomah et al.,^4^ Japiong et al.,^5^ World Health Organization,^9,36^ Dzwonczyk and Riha,^11^ Mavalankar et al.,^34^ Food and Drugs Authority Ghana^37^ and Bauserman et al.^38^

The introduction of medical devices, irrespective of their sources or initial costs, requires planning, information systems and knowledge of engineering and basic sciences.^39,40^ As such, we propose that the introduction of medical devices (whether through philanthropic activities, development partner agreements or direct purchase agreements) must be conceptualized within a single, enterprise-wide comprehensive acquisition and management (CAM) framework, as shown in Figure 3. This framework adopts elements from the published literature as indicated. ^4,5,9,11,34,36–38^ The CAM framework should be implemented by the Ministry of Health (representing hospitals and other public health care facilities) in collaboration with other relevant government agencies and funders.

**Figure 4.**
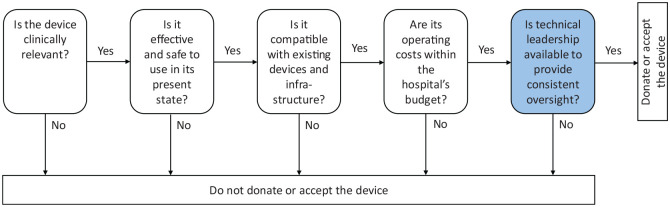
Elements of purposive funding for medical devices (a proposal). *Source*: World Health Organization,^9^ Dzwonczyk and Riha,^11^ Mavalankar et al.,^34^ Food and Drugs Authority Ghana^37^ and Bauserman et al.^38^

Rather than view the medical devices that they provide in isolation from or independent of the public health care system, funders should consider the devices as integral components of a system which consists of other medical devices acquired by other parties and limited human and financial resources.^36^ The CAM framework provides transparent, proactive mechanisms for transnational funders and recipients alike to assess devices for clinical relevance and effectiveness; safety, function and compatibility within the existing infrastructure; and financial and environmental impact. The CAM framework also enables hospitals to prepare for ancillary lifetime operating costs and empower hospital staff to make informed decisions about when to reject inappropriate devices and when to accept potentially useful devices. The enterprise-wide CAM framework for medical devices consists of the following key interdependent elements:

1. *Implementing clinical needs assessment* that is co-ordinated and involves an advocate who represents the interests of the recipient country.^11,36^ Findings from our study suggest opportunities to improve the needs assessment process. The needs assessment should consider whether a device is clinically relevant to the health care facility’s patient population and the skill sets of the clinical and technical staff. The process should include at least one end-user, a department lead, a technician or engineer, a head of administrative services of the facility and a representative of the funder. Every aspect of the CAM must be under the oversight of a technically skilled head of administrative services or executive at the facility level. To facilitate timely sharing and revision of the information, we recommend digitally documenting the information.^4,5^ Digital content may be more readily shared, accessed and updated than paper-based content.
2. *Defining appropriate specifications or requirements* for devices that address the expressed clinical needs. This element is best conducted under the purview of a clinical engineer or technician in conjunction with an end-user. The engineer or technician will outline the technical features of the device that will fulfil the clinical needs identified in the first step.
3. *Purposefully selecting devices* that meet or exceed the defined specifications, using pre-defined criteria (see Figure 4). The device must incorporate the elements of purposive funding for medical devices:
  - Is the device clinically relevant to the patient population and common indications presented at the proposed recipient’s hospital?^11,36^
  - Is the device effective (i.e. in working order) and safe to use (i.e. not expected to cause physical harm) in its present state?^9,36^
  - Is the device compatible within the existing network of devices and functional within the existing infrastructure (i.e. supply of electricity, water and human resources)?^36^
  - Are the device’s lifetime operating costs within the recipient’s budget – from acquisition to safe disposal – and will the existing supply chain accommodate the consumables?^36,40,41^
  - Is a qualified hospital executive available to provide technical leadership and consistent oversight?

For the device to be acquired, all the above questions must be answered in the affirmative.

4. *Enhancing procurement* through technical evaluation and coordination with installation, surveillance and disposal.^4^
5. *Providing* shipping and clearing expertise to expedite safe and reliable transportation.
6. *Implementing storage and distribution mechanisms* to minimize the time devices are exposed to uncontrolled temperature and humidity.
7. *Coordinating installation procedures* with procurement and monitoring.^40^ Monitoring refers to ongoing assessments and observation of the device’s performance.
8. *Coordinating operator training* to complement the existing and projected inventory of devices.^35^
9. *Ensuring sustainable functionality* by proactive surveillance of consumables, replacement parts and other ancillary resources.^11,38^
10. *Enforcing disposal mechanisms* that meet or exceed established regulatory standards and minimize environmental hazards. The Ministry of Health in collaboration with the Environmental Protection Agency can enforce disposal.

The first step in adopting the CAM framework is to institutionalize technical representation at the executive level of hospitals. The technical executive must have oversight capacity and must be trained to identify financial, technical and safety matters specific to medical devices and to determine mechanisms to address them.^39^ Engineers and biomedical scientists may fill this role. Introducing medical devices to hospitals before ensuring that there is a member of the executive (or management) team who is available and qualified to manage the devices is ill-advised.^32^ Introducing a technical leader on executive teams will require behavioural changes among hospital heads of administrative services – a challenge that must be addressed before equipping hospitals with medical devices.

The CAM framework will enable the Ministry of Health and hospital management teams to plan for requisite ancillary activities during initial discussions of initiatives rather than retroactively building a supply chain and training staff on a reactive and ad hoc basis. The CAM framework will ensure that transnational funders take ownership of safeguarding sustainability of their funded devices within the public health care system’s existing infrastructure.^11,33,34^

### Study limitations

The primary limitation of this study concerns recruitment of hospital staff for interviews. Three individuals located in central, administrative posts selected the hospital sites based on their personal judgements. All hospitals visited were owned by the Government of Ghana, and most of them were operated by faith-based entities. The perspectives of staff in hospitals operated by other organizations were not included. The second limitation was that informants primarily relied on their memories when relaying information about past experiences. There is recall bias associated with extracting data from individual memories. Nonetheless, the available information was presented coherently and clearly, showcasing the perspectives of a range of professionals associated with public health care delivery in Ghana. Triangulation of information from multiple data sources helped to mitigate the risk of recall bias.^42^ Third, while the national guidelines referred to donations of medical devices, transnational funding arrangements are often complex, involving some combination of philanthropy, long-term development goals, politics and financial gain. It was unclear whether the accounts of some key informants and the direct observation were linked to devices that were acquired through truly philanthropic activities (i.e. donations), transnational agreements involving elements of financial gain or other forms of reciprocity, or direct procurement using internally generated hospital funds. The fourth limitation concerns the representation of key informants. Funders and senior government officials were not represented in the interview sample. The richness of the data could have been improved with information from funders and senior government officials.

### Future research

Future research involving representatives of funders could provide insights into why funding organizations find it acceptable to provide medical devices to governments in resource-limited settings without clear policies in place. For funders that have self-enforced policies in place, research is needed to identify the factors that facilitate or hinder their implementation. Interviewing government officials may help policy makers to understand why the funding guidelines are not widely adopted in public hospitals.

## Conclusion

The study provides contemporary evidence of the factors that hinder sustainable access to medical devices in public hospitals as expressed through the words of frontline hospital staff and heads of administrative services in Ghana and as validated through direct observation and a review of guidelines. The study emphasizes the hospital staff’s need for sustainable models with identifiable milestones that can be used to track progress towards establishing maintenance capacity and ownership of externally funded medical devices. The study demonstrates the importance of prioritizing technical leadership on hospital management committees and developing a comprehensive acquisition and management framework for all medical devices, irrespective of their sources. The study also underscores the need for the Ministry of Health and transnational funders to acknowledge that some externally funded initiatives do not contribute to long-lasting access to medical devices. By accepting devices indiscriminately, the Ministry is itself creating a considerable burden on its health care system.

Policy makers, who represent the Ministry of Health and non-governmental agencies alike, can act upon the findings of this study to improve the value of transnational funding in general and access to medical devices specifically. Policy makers in other resource-limited settings may also find the results and framework described here useful. The Ministry of Health has an opportunity to strengthen its health care policies and implement social initiatives to ensure that access to donated devices are sustained such that health outcomes are enjoyed by the population.^13,43^ Specifically, hospital staff will benefit from updated and strengthened transnational funding guidelines, integrate them into a CAM framework for active surveillance and management of medical devices, and actively promote them within their facilities.

With renewed effort, the Ministry of Health, its hospital management teams, other frontline hospital staff and transnational funders can work together to truly facilitate sustainable access to medical devices in public hospitals.
